# Relationships between empathy and creativity in collective games: a comparison between handball and sitting ball

**DOI:** 10.3389/fpsyg.2023.1185462

**Published:** 2023-05-09

**Authors:** Alexandre Oboeuf, Sylvain Hanneton, Emmanuel Fernandes, Joséphine Buffet, Samantha Coquinos, Loïc Lecroisey

**Affiliations:** ^1^Université Paris Cité, Paris, France; ^2^Institut des Sciences du Sport et de la Santé de Paris (URP3625), Paris, France; ^3^Université de Picardie Jules Verne, Amiens, France; ^4^Centre Amiénois de Recherche en Education et en Formation (EA 4697), Amiens, France

**Keywords:** motor creativity, sociomotor empathy, instrumental empathy, socio-affective empathy, motor situation

## Abstract

In collective motor situations, creativity and empathy are central and strongly connected to cognitive and affective processes. Indeed, in the environment of high social uncertainty of games and sports, empathy would allow the player to anticipate motor behaviors in order to promote creative decision-making, i.e., to destabilize his opponents. On this basis, this study pursues two objectives. The first is to propose indicators to question the links between sociomotor empathy and motor creativity in an ecological situation. The second is to investigate the potential influence of the internal logic of two very different collective games (handball and Sitting ball) on the type of links that are woven between empathy and creativity. Two groups of students were recruited (*n* = 22 and 23) and participated in each of the games mentioned. The games were video recorded. The praxical communications made by each player were recorded and sorted by two trained observers. The results revealed major differences between the two studied collective games. In handball, there was a correlation between instrumental empathy (valuing cognitive aspects) and indicators of motor creativity (*p*  < 0.05). The more creative the players are (quantity, diversity and quality of performance), the more they manage to accurately anticipate the behavior of other players. In Sitting Ball, there was no correlation between creativity indicators and instrumental empathy. On the other hand, it is noticed that instrumental empathy was correlated with socio-affective empathy (*p*  < 0.001). To make their motor decisions, the players do not rely exclusively on the decoding of behaviors but significantly mobilize the feelings that they ascribe to the other co-participants. The results of this work invite reflection on the diversity of playful reading grids to be offered to students in order to develop their motor adaptability.

## 1. Introduction

In collective games, creativity and empathy are central and strongly connected to cognitive and affective processes (Parlebas, 1999; Jihyun et al., 2020; Oboeuf et al., 2020, 2022). First of all, creativity refers to the ability to generate new, original work that is meaningful in its context (Amabile, 1996; Runco and Jaeger, 2012; Anderson et al., 2014; Fardilha and Allen, 2020). During the game, participants are continuously adapting to the constraints of the internal logic by interpreting the behavior of their partners and their opponents (Parlebas, 1999; Oboeuf, 2010; Furley and Memmert, 2018). Teammates must be creative and unpredictable in order to individually and collectively thwart opposing projects (Oboeuf et al., 2009; Furley and Memmert, 2018; Rasmussen et al., 2019). This motor creativity is based on empathy, understood as the ability to put oneself in the place of others, i.e., to anticipate their feelings and/or their thoughts and/or their behaviors (Courchet and Maucorps, 1966; Berthoz and Jorland, 2004; Stanger et al., 2016). In the environment with high social uncertainty of games and sports (Márquéz Jiménez and Martínez de Santos, 2014), this empathetic mechanism would enable the player to pre-act, to anticipate behaviors in order to improve his creativity, in particular by increasing his ability to surprise opponents (Rasmussen et al., 2019; Oboeuf et al., 2020). In collective games, these two mechanisms are closely linked and yet there are few studies focusing on their interdependence. Therefore, our aim is to explore how empathy and creativity are intertwined in collective sporting games. First, it seems important to us to define more clearly the two central concepts involved.

## 2. Motor creativity and sociomotor empathy

### 2.1. The motor creativity

Motor creativity can be observed in an ecological situation (Oboeuf et al., 2020). To assess *in situ* the level of creativity of the players, it is necessary to know the structure of the communications that the players can use during the game. Indeed, creativity is dependent on the context, on the “frame” (Goffman, 1974), which channels motor behaviors and makes it possible to understand players’ strategic choices (Pic et al., 2018, 2020). We do not communicate in the same way during a basketball game or a dodgeball game (Parlebas, 1999; Collard, 2004; Pic et al., 2019), but the creative player is always the one who, within a given set of constraints, will succeed in putting the adversary in difficulty in his communication choices.

**Figure 1:**
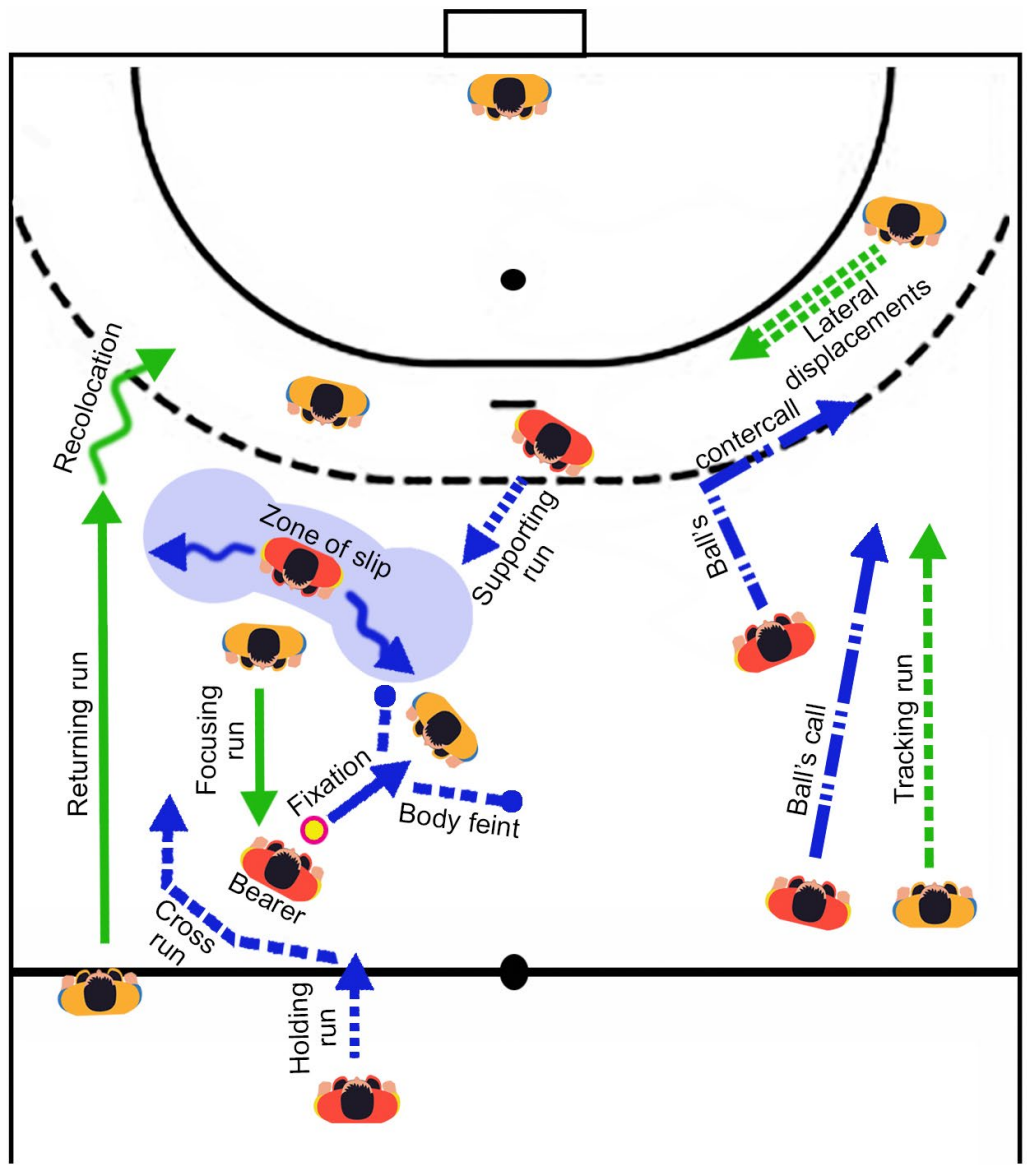
The sign system of handball. The main praxemes mobilized by handballers during the game. Two praxemes do not appear on the figure. (i) “Shooting feint”; (ii) “Passing feint.”

In team sports and traditional sporting games, praxical communication is divided into two main interdependent categories (Parlebas, 1999). The first concerns direct communication. This is often the only worthy of interest, because it is closely linked to the performance of the motor task: it involves a direct relationship to the object (pass, shot, interception, etc.) or to the body of the partner or of the opponent (contact, touch of capture, touch of delivery, etc.). This is the first-degree interpretation of the behavior of practitioners (Oboeuf et al., 2009). The second category concerns the signs (or praxemes^1^) which serve as a support for these direct communications and ensure the overall dynamics of the game: we name it indirect communication. In handball, the “focusing run” is a sign: if a player produces an approach run to reduce an opponent’s possibilities of action, the “running” behavior will be the signifier. The message or signified, meanwhile, will be the attempt to dissuade the player aimed at reducing his ability to move or to force him to separate from the ball. For a given game, all these praxemes are organized into a system (Figure 1). The analysis of this system of signs comes under the semiology of motricity, that is to say semiotricity (Parlebas, 1999; Bordes, 2020; Lavega-Burgués et al., 2022; Martínez-Santos et al., 2022; Parlebas, 2022). This semiotricity offers the opportunity to better understand the diversity of motor behaviors used by participants *in situ*. Understanding this communicational diversity is an issue but also a necessity to understand creativity in collective games. In effect, to be creative during the game, players must adapt to this system of signs, “this secret code” (Oboeuf et al., 2019, 2022), by assigning the right meanings to the behaviors of other players. *De facto*, mastering this code, made up of praxemes and their possible articulations, is an issue for promoting motor creativity (Oboeuf et al., 2020). In collective games, motor creativity means: (1) the ability of a player to mobilize a large number (fluency) and a large variability (flexibility) of direct and indirect communications (praxemes), i.e., to demonstrate praxical divergence. This is the quantitative side of creativity and (2) the ability to make the right motor decisions on a temporal sequence of play, i.e., to demonstrate praxical convergence. This corresponds to the overall ability of the player to propose diversified, elaborate, original but also adapted motor responses during the game (Memmert et al., 2013; Lubart et al., 2015; Zhu et al., 2019). This is the qualitative side of creativity. The creative player is able on the one hand, to energize the game by multiplying and diversifying the communications, and, on the other hand, to make the best possible decisions. He is the one who proves to be the most capable of weakening the opposing balance by proposing new responses adapted to the context in which they appear (Lubart et al., 2015; Furley and Memmert, 2018). It is important to specify that in a particular context, participants or groups that produce the most ideas or actions are often those that take the most adapted and original decisions (Lubart et al., 2013).

### 2.2. The sociomotor empathy

Players of collective games constantly make assumptions about motor behaviors. Some players shine in this area: they sometimes predict with a strong accuracy the future of the action in progress and influence its progress according to their projects. Sometimes they take the ball out from an opponent by “not falling” into the trap of his dribble. Sometimes they guess a pass line at the right time to intercept the ball. In other situations, they recover it with a skillful spatial positioning. Sociomotor games, in other words games with essential motor interactions (Parlebas, 1999; Collard, 2004), involve permanent anticipation. Each player is confronted with a large number of signs or praxemes (ball calls, cross runs, various feints, etc.) and must quickly make the best decisions. What does this adversary approaching me want to do? Should I pass the ball to my partner, who has just completed this cross run? And if an opponent anticipates this pass, would not it be better for me to dribble the one coming my way? The player thinks the other thinks he is going to act a certain way and even more than that, he thinks the other thinks he thinks! The anticipations of anticipations multiply on the playground… Behind these motor interactions are revealed fascinating and singular empathic mechanisms.

**Figure 2:**
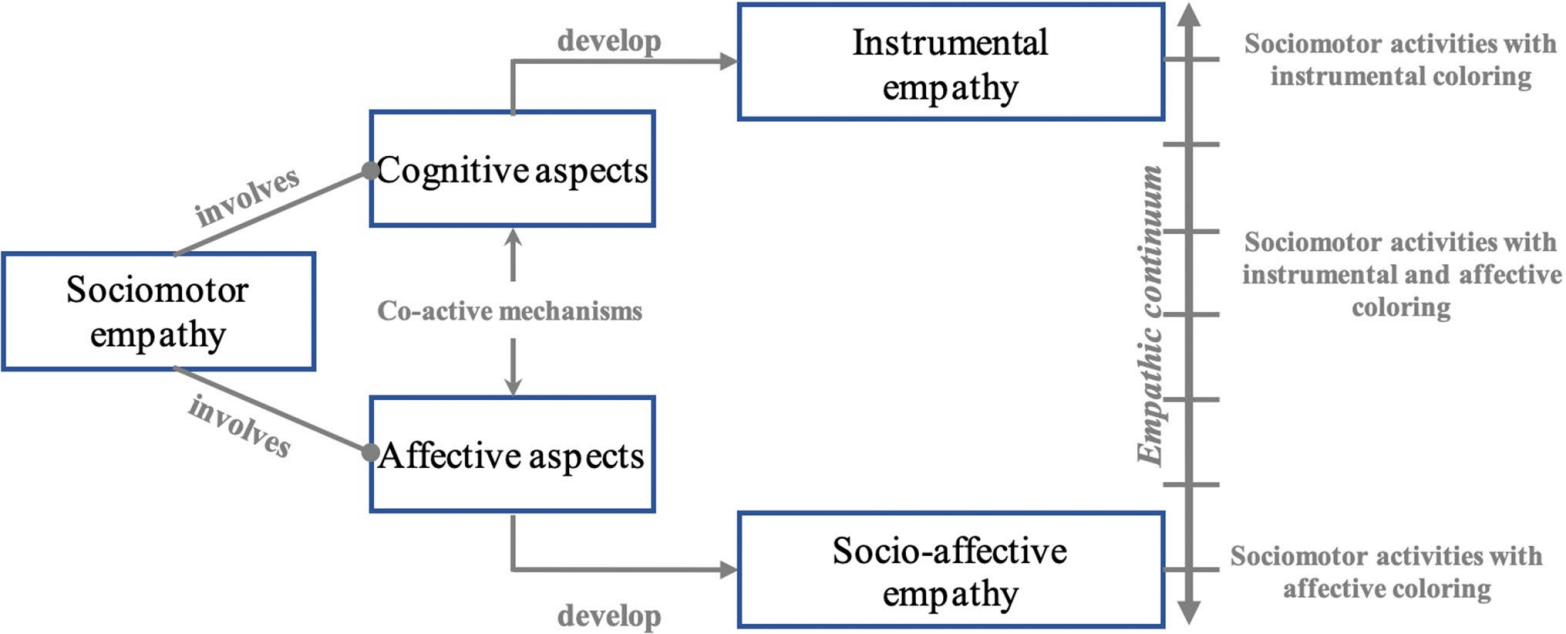
The two sides of sociomotor empathy. Empathy in sociomotor games is characterized by the co-activation of cognitive and affective mechanisms. The prevalence of one or the other of the corresponding empathy makes it possible to position each collective game on an empathetic continuum.

Parlebas advances the concept of sociomotor empathy, understood as “the process by which an interacting individual tries to grasp the point of view of another co-participant and takes it into account during his own motor behaviors of task resolution.” (Parlebas, 1999, p. 134). In activities that take place in the presence of others, this sociomotor empathy allows the player to adjust his motor decisions according to the projects he attributes to his partners and/or opponents. To operate this decentration, the player mobilizes cognitive and/or affective resources (Figure 2). The cognitive mechanisms feeding sociomotor empathy (memory, speed of information processing, attention, perception and reasoning in particular) helps the player to appreciate speeds, distances, support positions, tactics, strategic operations or to decode signs (praxemes or gestemes^2^). Thus, the motor decisions of the participants are nourished by observable elements: the player picks up clues grouped into signs to understand the behavior of others players. For example, in handball, if the player having the ball directs his steps (index 1), his shoulders (index 2) and his gaze (index 3) towards a direct opponent while accelerating sharply (index 4), it is said to make a “fixation” (praxeme). Cognitive mechanisms play a key role in the decoding mechanisms during the game.

At the same time, affective mechanisms participate in the decoding activity and come to weaken or reinforce our initial perceptions. The reading of the emotions of others and their interpretations (Damasio, 2010; Lecroisey, 2023), but also the pleasure felt, the desire for success or the motivational states perceived *in situ* are all determining factors in our decision-making. Obviously, the cognitive and affective aspects are always co-activated in all sociomotor situations. However, we are hypothesizing here that this joint deployment turns out to be very variable depending on the game. Depending on the modes of interaction with the partner(s) and/or the opponent(s), according to the spatio-temporal possibilities offered, according to the presence or absence of one (or more) object (s) mediating the confrontation, depending on the existence or non-existence of a scoring system generating or not a competitive challenge, the deployment of one or other of the empathic skills will be increased. All collective games cannot be placed in the same basket. Each internal logic orients and shapes differently the actions of the players (Parlebas, 1999; Lavega et al., 2014).

Concretely, certain sociomotor activities mobilize cognitive aspects to a greater extent. This could promote the development of instrumental empathy, which is close to what is called the theory of mind (Premack and Woodruff, 1978; Duval et al., 2011; Carlstedt and Balconi, 2019). The latter is a cognitive capacity that would make it possible to construct a representation of the mental states of other individuals independently of affective factors. In particular, it makes it possible to predict behaviors. In the games concerned, the affective aspects are not absent – they can never be, but they are reduced due to the need for success in the task (competitive stake). Here, it is mainly the mastery of the game code (the praxemes and their articulations) that allows the players to act effectively, and not the fact of knowing the affects of others. In other sociomotor activities, where the goal is not competitive and the modes of interaction with others are more flexible, the affective aspects can take a more important place. In this case, we anticipate the behavior of others by also relying on the knowledge we have of their feelings towards us. The affective resonance of these collective games would allow the player to mobilize a socio-emotional empathy. We defend the idea that there is a co-activation of these mechanisms with a more or less strong prevalence of one or the other according to the endogenous logic of the activity. Based on the elements mentioned above, it seems relevant to propose an empathic continuum where each collective game, according to its level of instrumental and affective coloring, could be place (Figure 2).

This study focuses on two distinct collective games (handball and Sitting ball) in order to: (1) study the mechanisms of instrumental and socio-emotional empathy and (2) to question the relationships between these empathic mechanisms and motor creativity. Well known, handball is a sport, in other words a motor situation codified in the form of competition, and institutionalized (Parlebas, 1999). It is a “strictly competitive game”: the final result decides between the winners and the losers. The need to win involves both being transparent to our teammates and concealing our plans from opponents. Attached to the expected gain of the game, time pressure forces on the players to permanently decode of a flow of motor behaviors. Thus, the internal logic of team sports would, in our view, favor the deployment of a rather instrumental empathy. Our opinion is that this is different in the traditional game of Sitting ball. It is a practice that has not received the institutional label. This game was chosen because it has no scoring systems and presents relational ambivalence and instability. This game can be considered as paradoxal. When I participate in a game of Sitting ball, there is no competitive stake and I can choose to cooperate or oppose the other participants according to my desires. Concretely, in Sitting ball, at a given moment, I can decide to pass or shoot the player facing me (ambivalence). Also, I can decide to pass the ball to a player that I have previously tried to “eliminate,” or to shoot at a player to whom I have previously made a pass (instability). The freedom offered to participants seems greater than in handball, where exclusivity and stability freeze relationships with partners and opponents. Praxical code appears as less complex in some traditional games, but freedom seems greater there. This is the reason why we postulate that the role of the affective side is amplified in these games. The absence of competitive stake and the possible interactions allow players to rely on the knowledge they have of the feelings of others to anticipate their behavior. Socio-emotional empathy can play a key role in this.

Based on the above considerations, the proposed study had two objectives:

1. To propose a method to assess instrumental empathy in collective game situations.
2. To reflect on the links between the sociomotor empathy and the motor creativity in ecological situation.

In connection with these objectives, two hypotheses were formulated:

1. It is possible to construct indicators to assess the instrumental empathy of players *in situ*.
2. The level of motor creativity is correlated either with instrumental empathy or with socio-affective empathy. In Sitting ball, motor creativity would rather be correlated with socio-affective empathy while in sports, it would be more associated with instrumental empathy.

## 3. Materials and methods

### 3.1. Study design

The use of mixed methods enables the study of the scenario of playful specificity due to the relevance of the temporal order of motor events (Arias-Pujol and Anguera, 2017). Mixed methods allow the integral vision of the object of study, the flexibility of the conceptual framework, and the inclusion of new dimensions (Johnson et al., 2007), which is suitable for the analysis of motor creativity and sociomotor empathy in motor situations. This choice is justified by the work on purely quantitative aspects (calculations of empathy indices, number/diversity of praxemes and data quality control) with the use of qualitative aspects such as the design of a grid observation (in order to identify the direct communications and praxemes of each collective game) and the convergence assessment by trained judges (Storme and Lubart, 2012). For this, the use of mixed methods was justified by observational methodology, based on the categories of communications and the temporal structure of motor actions. An observational methodology is a methodological approach adapted to work on the ecological dimension in sport and physical education (Pic et al., 2018). More concretely, a quadrant III observational methodology was applied (Anguera and Hernández-Mendo, 2016). A design was applied that was: (a) nomothetic, as data on different players were recorded, (b) punctual, because the observation were raised in a precise moment, and (c) multidimensional, since different dimensions (criteria) were taken into account.

### 3.2. Participants

To carry out this research, we recruited 2 groups of 22 and 23 students (*n* = 45) in the Faculty of Sports Sciences at the University of Picardie Jules Verne in Amiens, in the Hauts-de-France region (France). Among these 45 participants, there are 16 female students and 29 male students. These were 2nd year students (M: 19.3 years; SD = 1.53). The students chosen for the study were all specialists in team sports (basketball, football, handball, rugby, volleyball, field hockey). On average, experience in practice was 6.3 years (SD = 2.47). More concretely, our inclusion criteria are as follows: (1) be in the second year of a faculty of sports sciences and (2) be a specialist in team sports (at least 3 years of federal practice before the study). The exclusion criteria are as follows: (1) not being a specialist in team sports and (2) practice a collective sport at the federal level for less than 3 years. This research was conducted in accordance with the ethical principles of the Declaration of Helsinki and the recommendations of the ethics committee of Paris Descartes University (France). Notably, all students who participated in the study completed an informed consent document.

### 3.3. Procedure

The 2 groups of students specialized in team sports took part in 2 collective games: handball and Sitting ball. Each group participated, over two sessions, in each of the activities presented (30 min per game). Each of the 45 participants therefore played 60 min in each game over the two sessions. This represented 120 min of actual play per participant, i.e., 6,600 min to be analyzed for all players. The direct communications and praxemes of each participant were filmed, deciphered and sorted in order to calculate our indices of sociomotor empathy and motor creativity. Each part was recorded through the use of two cameras so that it was possible, in case of doubt among the judges, to resort to a second viewing angle. The recordings covered from the beginning to the end of the game. To carry out the data analysis, 20 students specialized in team sports and who did not participate in the study were trained in the recognition of the different communications in a game situation. This training contained 6 h of theoretical contributions and 10 h of practice. During the 6 theoretical hours, the students are presented with the different structural characteristics of collective games (ambivalent or exclusive network, stable or unstable, balanced or unbalanced, symmetrical or dissymetrical) but also the different categories of praxical communications (directs communications, praxemes and gestemes). The way in which praxical communications are articulated through the sequence of sociomotor sub-roles is also discussed. During the 10 h of practice, the students learn to spot the praxical communications and the sociomotor sub-roles of the players (videos) then will learn to spot, in pairs, the indicators of low creativity and high motor creativity. It is important to specify that in handball, there are 20 praxical communications (5 direct communications and 15 praxemes): shot, pass, interception, ball recovery, contact, recolocation, focusing run, supporting run, slip, ball’s call, ball’s countercall, holding run, cross run, fixation, lateral displacements, returning run, tracking run, body feint, shooting feint and passing feint. In Sitting Ball, there are 13 praxical communications (4 direct communications and 9 praxemes): shot, pass, interception, ball recovery, slip, ball’s call, ball’s countercall, approach run, recoil run, recolocation, body feint, shooting feint and passing feint. For this study, and for each actor, two observers were responsible for recording the communications of the actors *in situ* (fluidity and flexibility). The observers who note the data relating to the praxical divergence are not those who judge the overall performance (praxical convergence). In order to limit judgment biases, two other trained observers are in charge of this work.

### 3.4. Calculation of the three indices of motor creativity (FLU, FLEX and CONV)

To calculate the overall motor creativity index of each player, it is necessary to obtain a praxical divergence index and a praxical convergence index. For the first, we consider in each game the number of communications (fluidity) and the diversity of mobilized communications (flexibility). Praxical convergence, on the other hand, is an evaluation of the overall performance, that is to say of the player’s ability to make the right motor decisions. For each player, it was carried out by two trained judges who must assign a score between 1 and 10. The player close to 1 is considered to be not very creative: he makes decisions that never surprise his opponents and destabilize his partners. The player close to 10 is considered as very creative: he makes decisions that surprise his opponents and help his partners. We consequently obtained 3 indicators of motor creativity: praxical fluidity (FLU), praxical flexibility (FLEX) and praxical convergence (CONV).

### 3.5. Calculation of the instrumental empathy index (IEMP)

In order to obtain an index of instrumental empathy in games, we propose to mobilize the notion of sociomotor sub-role, understood as “the basic behavioral unit of the strategic functioning of a sports game” (Parlebas, 1999, p. 344): “Dribbler,” “Shooter,” “Passer,” “Receiver,” “Dissuader” or “Interceptor” are some examples of sub-roles in handball (Oboeuf, 2010). In reality, the sub-role is a label that qualifies a particular relationship of the player to others, to space, to time and to a possible object, i.e., to the internal logic. It refers to a class of motor behaviors that groups together actions judged to be equivalent from a strategic point of view. It should be noted that instrumental empathy is omnipresent in the participant’s motor decision-making, regardless of the sub-role assumed. However, we believe that certain sociomotor sub-roles are more closely linked to the empathic capacities of the player and that they are good indicators of it. In handball, we retain 3 sub-roles: “Dribbler,” “Interceptor” and “Recoverer.” If I recover the ball when my opponent tries to put me at fault, if I manage to give false information to my opponent in order to dribble him, or if I intercept the ball, I have significant empathetic acuity.

To calculate our Instrumental Empathy Index (IEMP), we summed the number of successful dribbles, recoveries and interceptions (r) and divided it by the number of attempted dribbles, recoveries and interceptions (t). This index evolves between 0 and 1 and it is maximum when r = t, i.e., when the player has succeeded in all the dribbles, recoveries and interceptions that he has attempted. In Sitting ball, we relied on the “Dodger” and “Shooter” sub-roles. The player who manages to hit his target manages to anticipate his movements, and the one who manages to dodge an opposing attempt guesses his opponent’s intentions. We can classify for each of the two games the players from the most to the least at ease in these exercises of sociomotor decentering. It then becomes possible to perform a correlation calculation between the indices of instrumental empathy and the three indicators of motor creativity (fluidity, flexibility and convergence).

### 3.6. Calculation of the socio-affective empathy index (SAEMP)

In order to question the place of the affective factors associated with empathy in the two chosen games, we ensured the passing of a sociometric questionnaire at the beginning of the first lesson. It seems relevant to us to carry out the administration of the questionnaire before the establishment of the collective games. Indeed, insofar as games are a social support conducive to the development of interpersonal relationships (Parlebas, 1999), it seems important to us to carry out a preliminary mapping of socio-affective relationships. This ensures the stability of the data in order to see how socio-affectivity influences the empathic mechanisms involved.

This sociometric questionnaire allows “both metric and clinical study of affective relationships and relationships of influence within groups or communities” (Parlebas, 1992, p. 22). It offers the opportunity to bring out socio-affective relationships within a group. In order to bring out these relationships, it is necessary to be attentive to the assessment criterion used for the 4 questions constituting the sociometric questionnaire (choices, rejections, expectations of choices and rejections). Indeed, if the assessment criterion is functional or instrumental, it is possible that respondents choose partners because of their skills in the activity concerned, and not because they like them. In our case, we ask the students who they would like to be with, but also not to be with, with a view to a discovery stage in Outdoor Physical Activities taking place at the end of the academic year. In this case, these are non-competitive activities only. This criterion makes it possible to obtain responses of great sincerity and to construct a map of affinity relations within the group (Parlebas, 1992). So we asked each individual to tell us who they would like to be with (choice), but also not to be with (rejections), in view of a situation of intense affective communication in the future. Beyond choices and rejections, the sociometric questionnaire also makes it possible to take an interest in the expectations of choices and rejections (Courchet and Maucorps, 1966; Parlebas, 1992; Oboeuf and Besombes, 2016). In other words, we also ask each student to give us the names of those who, in their opinion, will choose or reject them. These expectations will make it possible to operationalize the calculation of a socio-affective empathy index. Our interest is therefore focused on an index of self-empathy, which is the prediction of the designations of others in regard of oneself. It is a question of knowing to what extent each individual is aware of the choices and rejections of which he himself is the object. It is by comparing the perceptions of choices and rejections of each individual with the choices and rejections actually received that we obtain this index of empathy evolving between 0 and 1. We guess that the person who anticipated all the choices and rejections formulated towards him will be said to be empathetic (*n* = 1), while the one who struggles in this exercise will be said to be not very empathetic (*n* = 0).

Parlebas (1992) distinguishes two sub-indices, the average of which gives the empathy index: (1) Relational sensitivity: for each individual, it is the ratio between the number of perceptions that are exact (e) emitted by the subject (i.e., having been confirmed) and the total number of designations and rejections received (d) by the subject (s = e / d). This index varies between 0 and 1, s being maximum when e = d, i.e., when the subject has perceived all of the choices and rejections of others towards him and (2) Perceptual realism: for each individual, it is the ratio between the number of perceptions of the subject which are exact (e) and the total number of expectations of choices or rejections (a) that he has formulated (r = e / a). This index also varies between 0 and 1, r being maximum when e = a, that means when a subject will have had, for example, 4 perceptions of exact choices and rejections, and that he will have had no expectation not confirmed. Concretely, if a person has a relational sensitivity equal to 0.9 and a perceptual realism equal to 0.7, we obtain the average socio-affective empathy index (SAEMP) as follows: (0.9 + 0.7) / 2 = 0.8. With an index of 0.8, the respondent has a good empathy. Once this work is done, we can, for each group, classify all of our students from the most empathetic to the least empathetic at the socio-affective level. These rankings can then be related to the three indicators of motor creativity and to the rankings obtained for instrumental empathy.

## 4. Data quality

To determine the data quality (Márquéz Jiménez and Martínez de Santos, 2014), inter-observer reliability and validity tests were carried out. Once the observers had uploaded the video to the Lince program, they started to record the praxical communications, separately. To calculate the praxical fluidity and the praxical flexibility, as soon as the observer spots a praxeme or a direct communication from the player, he presses the corresponding button to record the information. Secondly, to calculate the index of instrumental empathy, he will identify the sub-roles updated by the participants (“Dribbler,” “Recoverer” and “Interceptor” in handball; “Shooter” and “Dodger” in Sitting ball) but also success or failure in the attempt (for dribbling, shooting and dodging). The Pearson and Spearman correlation coefficient were used. The values reached always exceeded values of 0.97, thus indicating a high correlation in inter-observer measurements. To assess convergence, for each player, two independent raters (trained) judged the overall performance. They were not informed about the objectives of the work carried out. The inter-judge reliability coefficient were above the critical limit of 0.80. Indeed, the inter-judge correlation coefficients were all above 0.95.

## 5. Variables

Three dependent variables are associated with creativity: praxical fluency, praxical flexibility and praxical convergence. Fluency (FLU) was defined as the sum of all direct and indirect communications used by a player. Flexibility (FLEX) represents the diversity of communications actualized by the player, independently of the number of occurrences. Convergence (CONV) corresponds to the average score assigned by the experts when evaluating the overall performance of each participant. Then there are two other dependent variables: the socio-affective empathy index and the instrumental empathy index. Socio-affective empathy (SAEMP) represents the ability of the participants to guess the choices and affective rejections of others participants. Instrumental empathy (IEMP) represents the ability of players to anticipate the motor behaviors of participants *in situ*.

## 6. Data analysis

Therefore, we obtained 5 dependent variables (FLU, FLEX, CONV, SAEMP and IEMP) with two factors: type of activity (HB or SB) and group membership (G1 or G2). These are our 2 independent variables. First, we studied the differences between our two groups using a Mann-Witney test. Then, Pearsons correlation tests were performed between our 5 dependent variables. Finally, in order to investigate more precisely what could be the relationships between the sociomotor empathy and the motor creativity we carried out two different factor analysis separately for the two activities. The significance level was set at 0.05. On this basis, we distinguish in our results different levels of significance: *p* < 0.05, *p* < 0.01 and *p* < 0.001.

## 7. The data repository

The data repository is saved in open csv format on the *research.data.gouv.fr*. site at the following DOI location: https://doi.org/10.57745/1C8TIM. This repository consists of 11 columns and 46 lines. The results of each student are presented line by line (lines 2 to 46) according to the indicators of creativity (fluidity, flexibility and convergence) and empathy (instrumental and socio-affective) presented in column (column B to E for the Sitting ball and F to I for handball). The result of socio-affective empathy for each student, obtained through the sociometric questionnaire, is in column J. Remember that fluidity (indicator of praxical divergence) represents, for one collective game, the number of praxical communications realized by each player (60 min per game). This result evolves between 33 and 432 communications in Sitting ball and between 76 and 611 communications in handball. Flexibility (indicator of praxical divergence) represents the diversity of communications used by each player. This result evolves between 4 and 10 communications (out of 13 possible) in Sitting ball and between 5 and 16 communications (out of 20 possible) in handball. The results of praxical convergence evolve between 1 (little creative) and 10 (very creative). This rating is awarded for each game by trained judges based on the overall creative performance of the player. Then, there are two indices of empathy. The first is instrumental empathy and is associated with the player’s ability to guess the choices of partners and opponents in a game situation. This result always evolves between 0 and 1 because it is a ratio between the number of anticipations attempted and the number of successful anticipations. It is minimum when the player succeeds in none of his motor anticipations (0) and maximum when he succeeds in all of them (1). The second corresponds to the socio-affective empathy index which also varies between 0 and 1. It is calculated from, on the one hand, the average of the ratio between the number of perceptions of choice and confirmed rejections and the choices and rejections actually received (relational sensitivity), and on the other hand, the relationship between the number of perceptions of choices and rejections confirmed and the perceptions of choices and rejections made (perceptual realism). No other processing was performed on the data before analysis.

## 8. Materials

Judges recorded the direct, indirect communications and sociomotors sub-roles with the Lince software (Gabín et al., 2012) and the JASP statistical software^1^ was used for statistical computation and analysis.

## 9. Results

### 9.1. The sample

Two groups of, respectively, 22 and 23 students participated in two collective games (handball and Sitting ball). First, we compared the mean of all the measures between the two groups to search for significative differences between them. Since the conditions for applying a t-test were not always fulfilled, we used Mann-Witney tests for the comparisons. None of the comparisons exhibited a significant difference. Consequently, we considered all the participants as a unique sample in the subsequent analyses.

### 9.2. Empathy and creativity measures

We then studied separately the relationship between empathy and creativity measures for the two collective games. Considering the Sitting ball activity, we did not find any correlation between the empathy measures (instrumental empathy and socio-affective empathy) and the creativity measures (praxical convergence, praxical fluidity and praxical flexibility). On the other hand, there was a significant correlation between the 3 indicators of creativity (Table 1): fluidity and flexibility (*p* < 0.001), fluidity and convergence (*p* < 0.001) but also flexibility and convergence (*p* < 0.001). The players who make the most communications were also those who diversify them the most. In addition, they were also those whose performances were considered most creative. Another interesting result is that there was a significant correlation (*p* < 0.001) between the two forms of empathy (Table 2). It seems that the players who best guess the feelings of others towards them (socio-affective empathy) were also those who best guess their behavior in the game situation (instrumental empathy).

**Table 1:** Pearson’s correlation test applied to the dependent variables for the game of Sitting ball.

	Pearson’s r	*p*
**FLU SB – FLEX SB**	**0.655** ^***^	***p* < 0.001**
**FLU SB – CONV SB**	**0.760** ^***^	***p* < 0.001**
FLU SB – IEMP SB	−0.016	0.915
FLU SB – SAEMP	−0.190	0.210
**FLEX SB** –**CONV SB**	**0.678** ^***^	***p* < 0.001**
FLEX SB – IEMP SB	0.060	0.694
FLEX SB – SAEMP	−0.107	0.485
CONV SB – IEMP SB	−0.159	0.296
CONV SB – SAEMP	−0.193	0.203
**IEMP SB – SAEMP**	**0.594** ^***^	***p* < 0.001**

^*^p < 0.05, ^**^p < 0.01, ^***^p < 0.001. The significant results are put in bold.

**Table 2:** Pearson’s correlation test applied to the dependent variables for the game of handball.

	Pearson’s r	*p*
**FLU HB – FLEX HB**	**0.619** ^***^	***p* < 0.001**
**FLU HB – CONV HB**	**0.601** ^***^	***p* < 0.001**
**FLU HB – IEMP HB**	**0.673** ^***^	***p* < 0.001**
FLU HB – SAEMP	−0.091	0.551
**FLEX HB – CONV HB**	**0.796** ^***^	***p* < 0.001**
**FLEX HB – IEMP HB**	**0.320** ^*^	***p* < 0.05**
FLEX HB – SAEMP	−0.008	0.956
**CONV HB – IEMP HB**	**0.301** ^*^	***p* < 0.05**
CONV HB – SAEMP	−0.140	0.358
**IEMP HB - SAEMP**	**−0.331** ^*^	***p* < 0.05**

^*^p < 0.05, ^**^p < 0.01, ^***^p < 0.001. The significant results are put in bold.

For handball, as for Sitting ball, there was a correlation between the three indicators of creativity (Table 2). Fluency was correlated with flexibility (*p* < 0.001), fluency with convergence (*p* < 0.001) and flexibility with convergence (*p* < 0.001). The results were not completely the same as for the handball activity since we found that the instrumental empathy was correlated with the three creativity measures (see Table 2). In other words, the more creative the players were (quantity, diversity and quality of performance), the more they managed to accurately anticipate the behavior of other players during the course of the game. Another result was the significant negative correlation between socio-affective empathy and instrumental empathy (*p* < 0.05). In other words, in handball, the participants who best guessed the behavior of others are those who present the lowest levels of socio-affective empathy.

To investigate more precisely what could be the relationships between empathies and creativity indexes we carried out two different factor analyses separately for the two activities. This factor analyses were done to search for independent dimensions in the variability of measures. The method used was the parallel method with oblique rotation (promax). The obtained factors were slightly different for the two activities (Table 3).

**Table 3:** Factors analysis for the Sitting ball (left column) and the handball (right column).

	Sitting ball		Handball	
	Factor 1	Factor 2	Factor 1	Factor 2
Convergence	0.864		0.877	
Flexibility	0.787		0.981	
Fluidity	0.860		0.560	
Instrumental empathy (IEMP)		1.015		0.993
Socio-affective empathy (SAEMP)		0.591		X

The factor analyses revealed two factors in both analyses. Considering the Sitting ball activity, empathy and creativity measures were separated in two factors. However, for the handball activity, only the instrumental empathy was represented in the model. The socio-affective empathy did not contribute significantly to any of the two dimensions.

## 10. Discussion

### 10.1. A strong footprint of the internal logic on the type of empathy mobilized

Complex cognitive and affective mechanisms are activated in collective games (Parlebas, 1999; Rasmussen et al., 2019). The presence of co-participants forces each player to interpret constantly renewed waves of direct and indirect communications to make their motor decisions. These decisions taken *in situ* are not the result of chance. They are based on subtle empathetic processes, i.e., on the player’s ability to guess the behaviors, thoughts and feelings of the other, of others (Berthoz and Jorland, 2004; Stanger et al., 2016). We formulated the hypothesis that to better understand the empathic mechanisms involved, it was necessary to consider the internal logic. For each game, the internal logic defines an interactional, spatial, temporal and material context that guides the ways in which players interact. According to the internal logic, the type of empathy mobilized would be distinct, with a more or less strong prevalence of cognitive or affective factors. In order to verify this postulate, indicators of empathy have been constructed, tested and validated by repeated observations.

For Sitting ball and handball, our trained observers were able to quantify and assess, for each player, his ability to anticipate the behavior of other players, i.e., his instrumental empathy (Oboeuf et al.; Oboeuf and Besombes, 2016). Thus, the Sitting ball player who succeeds in all of his shots and who manages to dodge all of the shots against him shows significant instrumental empathy. This is also the case, beyond technical considerations, of the handball player who succeeds in all his dribbles, the interceptions he tries and who recovers many balls. Our results revealed an interesting phenomenon. In the Sitting ball game, there was a significant positive correlation between instrumental empathy and socio-affective empathy (*p* < 0.001). Conversely, there was a significant negative correlation between these two forms of empathy in handball (*p* < 0.05). Concretely, in Sitting ball, knowing the feelings of others towards him seems to help the player to predict the behavior of the other participants. Socio-affective empathy is a facilitating factor. In handball, it could be a limiting factor. This is related to the fact that the internal logic of handball is binding at the relational level. Relations between players are said to be exclusive and stable (Parlebas, 1992; Oboeuf et al., 2020). Exclusive, because at some point, I cannot choose my partners and my opponents. Stable, because I cannot change partners and opponents during the game. To these constraints adds another determining characteristic: the competitive stake associated with the presence of a scoring system. In team sports, what counts is what counts (Parlebas, 1999). This is the reason why players are massively focused on decoding significant bodily indexes and mobilize the instrumental empathy (valuing cognitive aspects). The competitive stake of handball is opposed by the deeply relational stake of the game of Sitting ball. Its internal logic accentuates the freedom of the player. It is a playful reproduction of relational possibilities associated with our daily lives (Oboeuf et al., 2010). Relationships are said to be ambivalent and unstable. Ambivalent, because we can choose our partners and opponents at any given time according to our desires (pass or shot). Unstable, because you can change partners and opponents or the course of the game (shoot then pass or pass then shoot). The absence of a competitive stake reinforces the socio-affective hue of the Sitting ball.

If in team sports, the stake supplants the game, this does not seem to be the case with the game of Sitting ball. What are the consequences of these observations on the mechanisms of motor creativity activated in these two games? How does the socio-affective empathic coloration of Sitting ball influence the creativity of players? And the rather instrumental empathy of handball?

### 10.2. Contribution of sociomotor empathy to creativity process

Generally speaking, there are few studies on the articulation of empathy and creativity (Jihyun et al., 2020). Research on games and sports is no exception to this finding (Parlebas, 1999; Oboeuf et al., 2022). This is the reason why we proposed to compare indicators of motor creativity in an ecological situation (Oboeuf et al., 2020) with indicators of sociomotor empathy themselves linked to the game situation (Oboeuf et al., 2010). The results reveal major differences between the two studied collective games. In handball, there is a correlation between instrumental empathy and the three indicators of creativity: praxical fluidity (*p* < 0.001), praxical flexibility (*p* < 0.05) and praxical convergence (*p* < 0.05). The players who realize a lot of communications (fluidity) and diversify them the most (flexibility) are the participants with the highest instrumental empathy indexes. They are also those whose overall performance was assessed the most creative by expert judges (convergence). Even if these results cannot be generalized, they underline the strong interdependence that is woven between empathy and creativity in team sports. The more creative the players are (quantity, diversity and player match quality), the more they manage to accurately anticipate the behavior of other players.

Previously discussed, the internal logic of handball explains this result. In this environment of high social uncertainty, it is necessary to anticipate and pre-act by wisely decoding the behavior of co-participants (Martínez de Santos, 2007; Ghannouchi et al., 2019). Instrumental empathy is the key to creativity. As Winkin suggests, “if the observer has grasped the interactional system that governs the game of the participants, he can foresee the movements moments before their actual occurrence” (Winkin, 2001, p. 120). It is this time in advance that allows the player to put his opponents in difficulty. It is this time in advance that lets him to express his motor creativity. In Sitting ball, as in handball, the three creativity indicators are correlated. Fluid players are the most flexible and are also those realized the best performances (*p* < 0.001). These results corroborate those obtained in the literature (Lubart et al., 2015; Weiss and Wilhelm, 2022). However, unlike handball, there is no positive correlation between the three indicators of creativity and instrumental empathy. To make their motor decisions, the players also rely on the feelings they ascribe to the other co-participants rather than exclusively on the behaviors they mobilize. This is why instrumental empathy is correlated with socio-affective empathy (*p* < 0.001). This does not mean that the links between empathy and creativity are non-existent in this game. But the internal logic and the freedom it offers to the players undoubtedly involve the joint mobilization of social creativity (Van Bezouw et al., 2021) and motor creativity. It could be interesting to compare the results of social creativity tests (Lebuda and Glăveanu, 2019) with the empathy indicators constructed in this study. If the co-activation of the two empathic mechanisms is obvious, one however notes a more or less strong prevalence of one of the two mechanisms according to the endogenous logic of the activity.

Among the limitations of the study, it should be mentioned that the number of participants (*n* = 45) could have been larger. It would be interesting to carry out the same study with more groups and/or populations of different ages to see if the obtained results are weakened or confirmed. The influence, at a given age, of the level of physical, cognitive and affective development of participants can play a major role in the obtained results. It could also be interesting to question the influence of the internal logics of Sitting ball and handball on the development of socio-affective empathy. With this in mind, a sociometric post-test should be carried out to see: (1) whether these practices influence the evolution of this form of empathy and (2) which of these two games contributes the most to its development. Then, as we suggested, it would without a doubt be relevant to compare other forms of creativity (social, verbal, graphic, etc.) with the sociomotor empathy studied indicators. Each game, according to its internal logic, undoubtedly offers participants the opportunity to express one part or another of their creative potential. Finally, to better define the mechanisms involved in the strategic choices made *in situ*, we could conduct self-confrontation interviews with the players (Martinent et al., 2015). Such interviews could provide interesting elements to pedagogically support the development of sociomotor empathy and motor creativity.

### 10.3. Conclusion: empathy at the service of adaptability

Everyone reads the world through the window of their experiences. As such, the human brain can be described as projective, meaning “it projects its rules of analysis, its preperceptions onto the world, it is a generator of hypotheses” (Berthoz and Jorland, 2004, p. 257). At our level, these finding questions the motor experiences that students must face in Physical and Sport Education. What motor adventures should be favored to participate in the construction of a more “empathetic” world, potentially favorable to living better together? Should team sports be valued exclusively? On the other hand, should we offer traditional games with more flexible interactional modalities? Resolutely, our opinion is that it is necessary to diversify the reading proposed grids. Empathy is more than understanding other people’s feelings or anticipating their behaviors (Courchet and Maucorps, 1966; Decety and Ickes, 2009). Empathy in handball is mainly based on the decoding of significant bodily indexes, while in Sitting ball the feelings that we attribute to others take a major place. Empathic acuity, its deployment and development are jointly based on affective and cognitive factors. Empathy is a subtle blend of these two types of resources. To swap one’s “egocentric” frame of reference for an “allocentric” frame of reference (Berthoz and Jorland, 2004; Gauthier and Berthoz, 2019), it is therefore necessary to go through a diversity of motor experiences. We can think that the more they diversify, the more we broaden the spectrum of our reading grids… and the more we adapt to the random flow of our social interactions with accuracy.

In this regard, it is undoubtedly crucial not to overvalue the cognitive aspects to the detriment of the affective aspects. The empathy must not be sclerotic, trapped in the pursuit of strictly rational and selfish purposes. It allows also to open a breach where the affective aspects can rush in order to reveal that “true altruism is not always reduced to a form of disguised selfishness” (Ricard, 2013, p. 14).

## Data availability statement

The raw data supporting the conclusions of this article will be made available by the authors, without undue reservation.

## Ethics statement

The studies involving human participants were reviewed and approved by the ethics committee of Paris Descartes University (France). The patients/participants provided their written informed consent to participate in this study.

## Author contributions

AO, SH, EF, and JB have contributed to the theoretical and methodological development of the manuscript, and prepared the results and discussion. SH, SC, and LL have contributed with the data analysis. All authors contributed to the article and approved the submitted version.

## Conflict of interest

The authors declare that the research was conducted in the absence of any commercial or financial relationships that could be construed as a potential conflict of interest.

## Publisher’s note

All claims expressed in this article are solely those of the authors and do not necessarily represent those of their affiliated organizations, or those of the publisher, the editors and the reviewers. Any product that may be evaluated in this article, or claim that may be made by its manufacturer, is not guaranteed or endorsed by the publisher.

## Footnotes

^1^The praxeme is a "motor behavior of a player interpreted as a sign whose signifier is the observable behavior and the signified the corresponding tactical project as it has been perceived" (Parlebas, 1999, p. 260).

^2^The gesteme "corresponds to the attitudes, gestures, facial expressions, motor behaviors that are performed with the aim of transmitting either a request, or an indication, or a tactical or relational injunction by simple substitution for speech" (Parlebas, 1999, p. 155).
